# IL-4 and IL-13 Differentially Regulate TLR-Induced Eosinophil-Basophil Differentiation of Cord Blood CD34^+^ Progenitor Cells

**DOI:** 10.1371/journal.pone.0100734

**Published:** 2014-06-27

**Authors:** Pia Reece, Gail M. Gauvreau, Roma Sehmi, Judah A. Denburg

**Affiliations:** 1 Division of Clinical Immunology & Allergy, McMaster University, Hamilton, Ontario, Canada; 2 Asthma Research Group, Firestone Institute for Respiratory Health, McMaster University Hamilton, Ontario, Canada; University of Cambridge, United Kingdom

## Abstract

Intrauterine environmental exposures have been shown to influence neonatal immunity and subsequent allergic disease development. We have previously shown that fewer lipopolysaccharide (LPS)-stimulated eosinophil-basophil (Eo/B) colonies grow from cord blood (CB) of high-atopic risk infants, compared to low-atopic risk infants. In the present study, we investigated whether a surrogate *ex vivo* T_H_2 milieu (i.e., either IL-4 or IL-13) could represent an underlying mechanism to explain our previous findings. CB CD34^+^ cells from healthy donors were cultured with IL-4 or IL-13 (in combination with LPS) and assessed for Eo/B differentiation using methylcellulose cultures and flow cytometry for related intracellular signalling pathways. Pharmacological inhibitors were added to the methylcellulose cultures to determine the effect of blocking intracellular signalling in CB CD34^+^ cells in relation to Eo/B colony forming unit (CFU) formation. Stimulation of CD34^+^ cells with IL-4, but not IL-13, reduced Eo/B CFU formation in the presence of LPS; this was found to be dependent on IL-4Rα and not IL-13Rα1. Additionally, IL-4 reduced the expression of ERK 1/2 after LPS stimulation, which was recovered by inhibition of IL-4Rα. While IL-13 did not have an inhibitory effect on ERK 1/2 expression, inhibition of ERK 1/2 significantly reduced Eo/B CFU formation. Thus, the responsiveness of CB CD34^+^ progenitor cells to LPS is differentially regulated by the T_H_2 cytokines, IL-4 and IL-13. This may have implications for *in utero* interactions between placental-derived pro-allergic cytokines and neonatal progenitor cells influencing Eo/B-mediated inflammatory responses in early life.

## Introduction

The dramatic and recent rise in allergies, along with their early onset suggests that *in utero* events are critical to the development of allergies [1]. Environments rich in microbes, such as farming environments, appear to protect against the development of allergies in children, especially when the exposure is pre-natal [2]. These protective effects are associated with alterations in both the neonatal innate [3], [4] and adaptive [5] immune systems. These studies suggest that the microenvironment of the uterus plays a key role in shaping the infant's response to environmental stimuli, which subsequently influences the development of allergy [1]. Although it is unknown how the maternal environment may exert such effects, it is tempting to speculate that the fetal immune system interacts with the cytokine milieu prevailing in the mother through the fetal-placental interface [6].

Our group has extensively investigated the role of hematopoietic progenitor cells in infant CB in relation to allergic risk and development of disease [7]–[10]. We have recently shown that the presence of maternal atopy alters CB progenitor toll-like receptor (TLR) phenotype and function; at-risk infant CD34^+^ cells express reduced TLRs with muted LPS-induced Eo/B CFU [10], compared to low-risk infants. Since LPS can induce Eo/B CFU from CD34^+^ cells via autocrine activation of MAPK [11] and atopic at-risk infants have elevated T_H_2 cytokines in their CB [12], [13], we were interested in what effect these cytokines may have on LPS-induced Eo/B CFU [10]. Relatedly, maternal cytokines (which can be transferred to the CB) have been shown to play instructive roles in fetal immune development. For example, increased maternal T_H_2 cytokines relate to both neonatal IgE production [14] and T regulatory cell numbers [6]. Additionally, there are strong correlations between maternal placentally-derived and CB-derived cytokine production [15]. Therefore, with the ability of maternal factors, such as cytokines in the intrauterine environment [15], to alter neonatal immune responses [6], we investigated the effect of a prototypical atopic T_H_2 milieu on hematopoietic progenitor cell responses to LPS.

The T_H_2 cytokines IL-4 and IL-13 are secreted by a variety of leukocytes and play an important role in the development of allergic responses. These cytokines are involved in IgE production [16] and eosinophil recruitment to the airways [17]. The expression of IL-4 is increased in the airways of allergic subjects [18] and in the CB of at-risk infants who subsequently develop atopic disease [12], [13]. Although these cytokines have recently been shown to influence human CB CD34^+^ cell chemotaxis [19] and murine bone marrow (BM) Eo/B CFU formation *ex vivo* [20] it is unclear what effects IL-4 or IL-13 might have on LPS-induced Eo/B CFU formation from human CB CD34^+^ cells.

In this study, we investigated whether the T_H_2 cytokines IL-4 and IL-13 influence LPS-induced Eo/B CFU of CB progenitor cells. We *hypothesized* that TLR-induced signalling may be altered by T_H_2 cytokines, representative of an “atopic milieu”, resulting in reduced Eo/B CFU [10]. In fact, we demonstrated that IL-4:IL-4Rα inhibits LPS-induced Eo/B CFU by blocking ERK 1/2 signalling in CB CD34^+^ cells. Since Eo/B differentiation is altered in children at risk for allergy [7]–[10], improved understanding of Eo/B differentiation processes may permit novel approaches targeting the regulation of these cells and the modulation of Eo/B-mediated allergic inflammation in early life.

## Materials and Methods

### Ethics statement

Pregnant mothers admitted to the Labour and Delivery ward at McMaster University Medical Centre, Hamilton, ON, Canada provided written consent for CB donation prior to delivery. This study was approved by the Hamilton Health Sciences/McMaster Faculty of Health Sciences Research Ethics Board.

### Cord blood collection

The CB samples were collected from otherwise healthy pregnant women. Upon delivery, each CB sample was collected in a 60 mL syringe containing 2 mL of heparin (1000 units/mL; Sigma, Mississauga, ON) and stored at 4°C until processing.

### Cord blood processing and CD34^+^ cell enrichment

CB samples were depleted of erythrocytes using gravity sedimentation as previously described [10]. To enrich the sample for CD34^+^ cells, mononuclear cells from the centrifuged pellet were selected using the EasySep Negative Selection Human Progenitor Cell Enrichment Cocktail with CD41 depletion (Stem Cell Technologies) as previously described [11]. The purity of CD34^+^ cells was between 85–90%.

### Methylcellulose cultures

Methylcellulose colony assays were grown for 14 days as previously described [10] using enriched CB CD34^+^ at a plating concentration of 2×10^4^ cells/35 mm×10 mm culture dish (Falcon Plastics, CA) in duplicate [11]. In addition to growth factors IL-3 (1 ng/ml), IL-5 (1 ng/ml) and GM-CSF (10 ng/ml) and the endotoxin LPS (10 µg/ml), cultures were supplemented with IL-4 or IL-13 (50 ng/mL) to assess their role in Eo/B CFU formation. For receptor blocking/neutralization experiments, anti-IL-4Rα or -IL-13Rα1 (5 and 50 µg/mL; R&D Systems) were added to the methylcellulose cultures supplemented with IL-4, LPS and hematopoietic cytokines.

The role of LPS-induced signalling on Eo/B CFU formation was investigated by adding the specific inhibitors to the methylcellulose culture assay: 5 or 50 µM PD98059 (ERK 1/2 inhibitor) [21], or 2 or 20 µM SB203580 (p38 MAPK inhibitor) [22] (Calbiochem, Cambridge, MA) or DMSO vehicle control. These concentrations were found to be non-toxic to cells [11].

### Phospho-flow to detect intracellular activation of signalling pathway molecules

**Figure 1:**
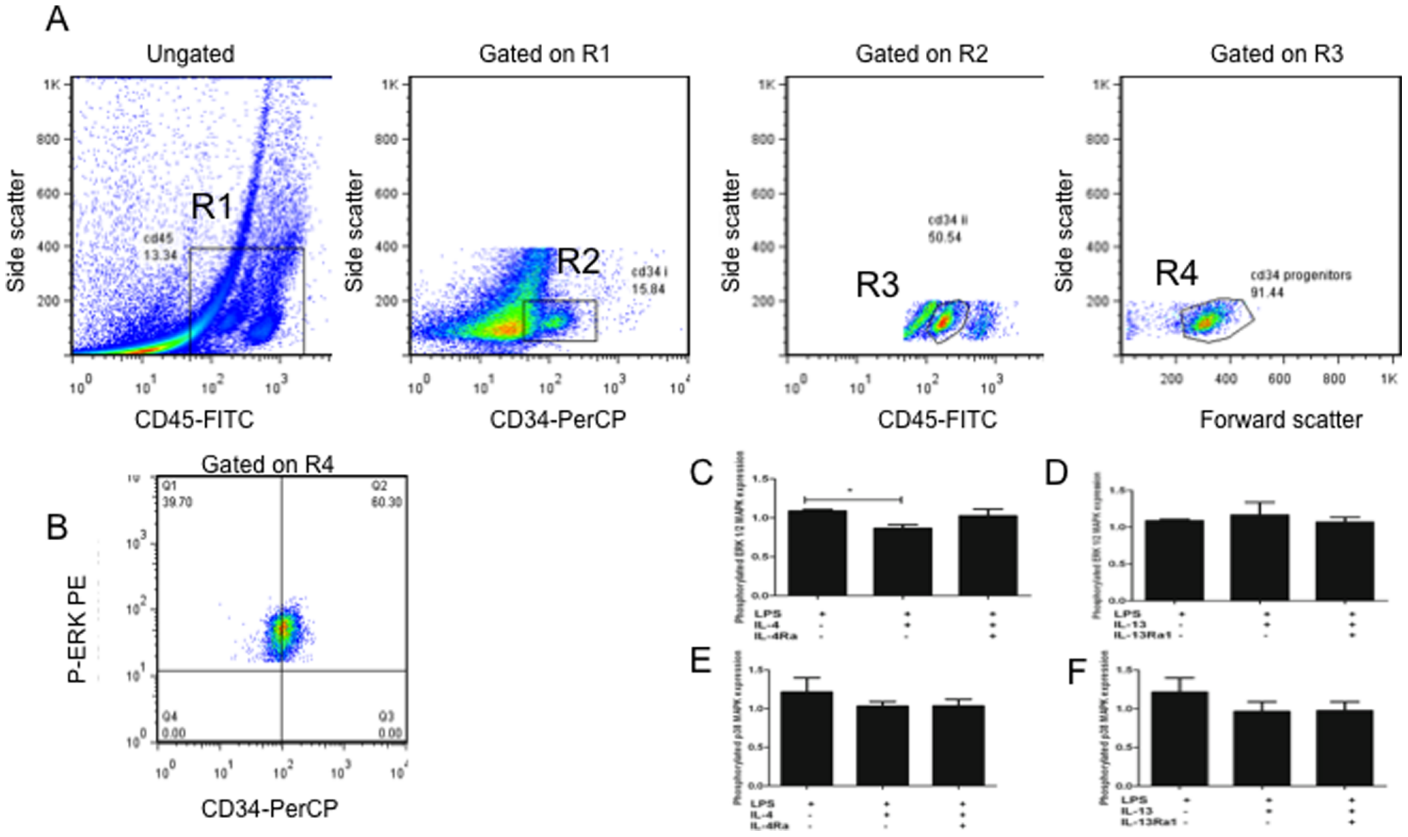
IL-4, but not IL-13, inhibits LPS-induced ERK 1/2, but not p38 MAPK. CD34^+^ cells were analyzed using phospho-flow cytometry with a sequential multiparameter gating strategy. A. Enumeration of CD34^+^ progenitor cells in cord blood. Enriched CB CD34^+^ cells were stained with CD34-PerCP and CD45-FITC and analyszed by flow cytometry. Numbers of true CD34^+^ blasts were determined by expressing cell numbers in R4 as a percentage of cell numbers in R1. Nonspecific staining with isotype controls were set at 2% in all experiments. A representative experiment is shown. B p-ERK expression on CD34^+^ cells (gated on the R4). A representative experiment of ERK staining is shown. Quadrant markers were set such that 2% or less of cells stained with the isotype control. C. CB CD34^+^ cells were pre-incubated with anti-IL-4Rα (C,E) or anti-IL-13Rα1 (D,F) Abs and stimulated with LPS and IL-4 (C, E) or IL-13 (D,F) respectively. Cells were stained with antibodies to intracellular ERK 1/2 (C,D) or p-38 (E,F) and analyzed using flow cytometry. A bar chart of the MFI expression index data is displayed for IL-4 (C,E) and IL-13 (D,F) data. Data are presented as mean ±SEM of 4 experiments and significant findings were determined using ANOVA with post hoc Dunnett comparison and presented by an asterisk (*), p<0.05.

LPS from Escherichia coli 0111:B4 was purchased from Sigma and used at the optimal concentration of 10 µg/ml as used previously [10]. Enriched CD34^+^ cells were stimulated with LPS and IL-4 or IL-13 (50 ng/mL) for 5 min to induce p38 MAPK expression, or 30 min to induce ERK 1/2 expression at 37°C 5% CO_2_ in Multiwell (Falcon) tissue culture plates with RPMI complete (RPMI plain, HEPES, Pen/Strep and FBS). These times were found to increase the expression of these respective proteins in CB CD34^+^ cells stimulated with LPS from our previous kinetic evaluations [11]. For receptor neutralization studies, cells were pre-incubated with anti- IL-4Rα or IL-13Rα1 (R&D Systems) for 15 min before the addition of IL-4 or IL-13 and LPS. Following the incubation, cells were analyzed using phospho-flow cytometry [11] with a sequential multiparameter gating strategy that has previously been shown to accurately enumerate CD34^+^ progenitor cells (Figure 1A) [7]–[10]. CD34^+^ blast cells (R4), are identified as cells with high expression of CD34, low to intermediate expression of CD45, and scatter properties characteristic of lymphoblastic cells. Briefly, cells were fixed using PhosFlow CytoFix Buffer (BD Biosciences, Ontario, Canada), and then centrifuged for 10 min at 1500 RPM. After washing, cells were permeabilized (PhosFlow Perm Buffer III; BD Biosciences) for 30 min on ice, washed with FACS Buffer (PBS, 0.1% sodium azide) then stained with a PE-conjugated phospho-specific mAb against p38 MAPK (pT180/pY182) or ERK 1/2 (pT202/pY204), FITC conjugated CD45 and PerCP conjugated CD34 or isotype control, all purchased from BD Biosciences [11].

### Acquisition and analysis

Acquisition was performed using a LSR II flow cytometer (BD Bioscience); 5×10^3^ events were collected for analysis. To enumerate CD34^+^ cells, we used an established multiparameter gating strategy as previously described [10]. The expression of phosphorylated ERK 1/2 or p38 MAPK was calculated using an expression index of the specific median fluorescence intensity (sMFI): sMFI_stimulated cells_/sMFI_unstimulated cells_ [23].

### Statistical analysis

Data were analyzed using IBM SPSS Statistics version 20.0 (Chicago, IL) and represented in figures as mean ± SEM for bar charts or as histograms for MFI data. Data that were not normally distributed were log transformed and subsequently analyzed with the ANOVA with a Dunnett *post hoc* analysis for many groups. We further applied the Mann-Whitney U test to assess the sensitivity or robustness of the results, and the results were consistent. We set the criterion for statistical significance *a priori* at α = 0.05. All p values were reported to three decimal places.

## Results

### IL-4 and IL-13 differentially influence GM-CSF- and IL-3-responsive Eo/B CFU formation after LPS stimulation

**Figure 2:**
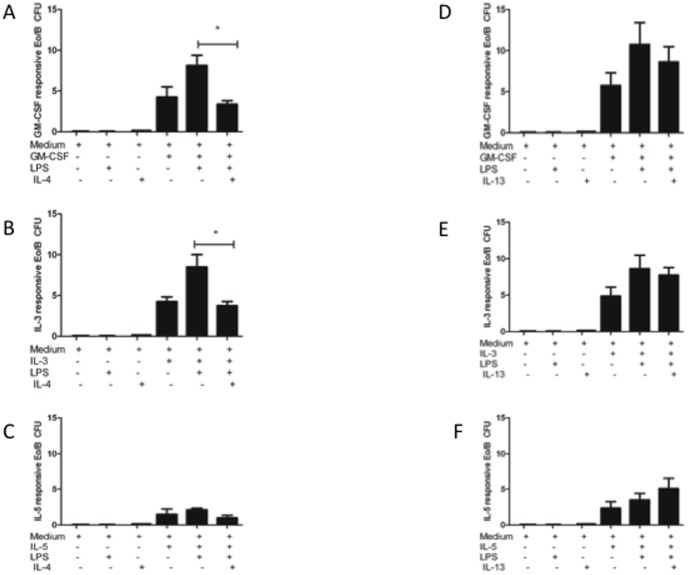
IL-4, but not IL-13, inhibits LPS-induced Eo/B CFU. CB CD34^+^ cells were cultured for 14 days (5% CO_2_, 37°C) with hematopoietic cytokines (A,D) GM-CSF (10 ng/mL) (B,E) IL-3 (1 ng/mL) (C,F) IL-5 (1 ng/mL), or with or without LPS, (A–C) IL-4, or (D-F) IL-13 (B). Eo/B cultures are tight, granular clusters of 40 cells or more Data are represented as mean ±SEM of 4 experiments and significant findings were determined using ANOVA with post hoc Dunnett comparison and presented by an asterisk (*), p<0.05.

We were first interested in whether the T_H_2 cytokines, IL-4 and IL-13, influence LPS-induced Eo/B CFU formation [11]. To test this, IL-4 and IL-13 were added separately to methylcellulose cultures of CB CD34^+^ cells containing hematopoietic cytokine and LPS. As shown in Figure 2A–F, incubation with IL-4 or IL-13 alone was not sufficient to induce Eo/B CFU production. However, the addition of IL-4 to GM-CSF + LPS (p = 0.028) or IL-3+LPS (p = 0.007) significantly decreased Eo/B CFU compared to these same cultures without IL-4 (Figure 2A–B). Conversely, the addition of IL-13 to any of the cultures with growth factors + LPS did not change the number of Eo/B CFU (Figure 2D–F). As observed in Figure 2C, we did not see significant alterations in IL-5-induced colonies. IL-5 cultures were included in this study as a control, since it is known that Eo/B CFU from IL-5 stimulated cultures are less abundant than CFU from IL-3 or GM-CSF stimulated cultures. Given the low numbers of colonies induced by IL-5, we were underpowered to investigate these comparisons.

### IL-4Rα is necessary for IL-4-induced reduction in GM-CSF- and IL-3-responsive Eo/B CFU

**Figure 3:**
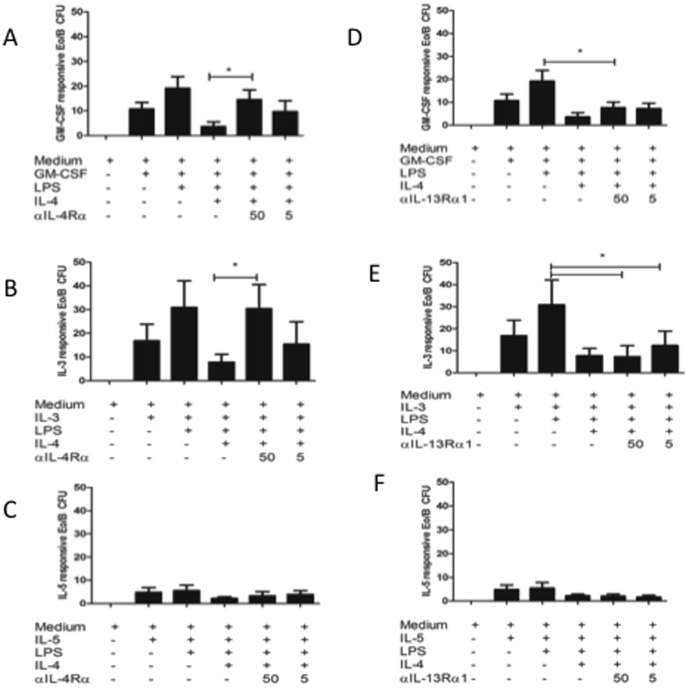
Inhibition of IL-4Rα, but not IL-13Rα1, recovers LPS-induced Eo/B CFU. CB CB CD34^+^ cells were cultured for 14 days (5% CO_2_, 37°C) with hematopoietic cytokines (A,D) GM-CSF (10 ng/mL), (B,E) IL-3 (1 ng/mL), (C,F) IL-5 (1 ng/mL), or with or without LPS, IL-4 and (A–C) anti-IL-4Rα, or (B–F) anti-IL-13Rα1. Eo/B cultures are tight, granular clusters of 40 cells or more. Data are represented as mean ±SEM of 5 experiments and significant findings were determined using ANOVA with post hoc Dunnett comparison and presented by an asterisk (*), p<0.05.

To determine whether the inhibitory effect of IL-4 on LPS-induced Eo/B CFU formation (Figure 2A–B) was dependent on IL-4Rα, CB enriched CD34^+^ cells were incubated with anti-IL-4Rα or anti-IL-13Rα1 in the methylcellulose cultures for 14 days. As shown in Figure 3A–B, the addition of anti-IL-4Rα to the cultures restored the number of GM-CSF- (p = 0.015) and IL-3-responsive (p = 0.033) Eo/B CFU formed in the presence of LPS +IL-4. Furthermore, the addition of anti-IL-13Rα1 did not restore the number of GM-CSF- or IL-3-responsive Eo/B CFU formed in the presence of LPS+IL-4 (Figure 3D–E); colony counts remained significantly reduced (p = 0.020 and p = 0.040 respectively) as compared to LPS stimulated cultures. This supports a specific role for IL-4:IL-4Rα signalling, independent of IL-13Rα1, in the inhibition of LPS-induced Eo/B CFU.

### IL-4 and IL-13 differentially influence LPS-induced phosphorylation of ERK 1/2

In order to investigate the potential mechanism of reduced LPS-induced GM-CSF- and IL-3-responsive Eo/B CFU formation in the presence of IL-4, we next determined if IL-4 had any effect on LPS-induced intracellular signalling [24] in CB CD34^+^ cells. To do this, CD34^+^ cells were incubated with LPS with or without IL-4 or IL-13 and analyzed using flow cytometry. As show in Figure 1C, the addition of IL-4 to LPS-stimulated CD34^+^ cells resulted in a significant reduction in the expression of ERK 1/2 (p = 0.040), whereas the addition of IL-13 did not (Figure 1E). Additionally, pre-incubation with anti-IL-4Rα to block IL-4 signalling recovered the expression of ERK 1/2 in the presence of LPS (Figure 1C). We were unable to detect any differences in the expression of phosphorylated p38 MAPK with the addition of IL-4 to LPS-stimulated CD34^+^ cells (Figure 1D).

### Inhibition of ERK 1/2 reduces GM-CSF-and IL-3-responsive Eo/B CFU formation after LPS stimulation

**Figure 4:**
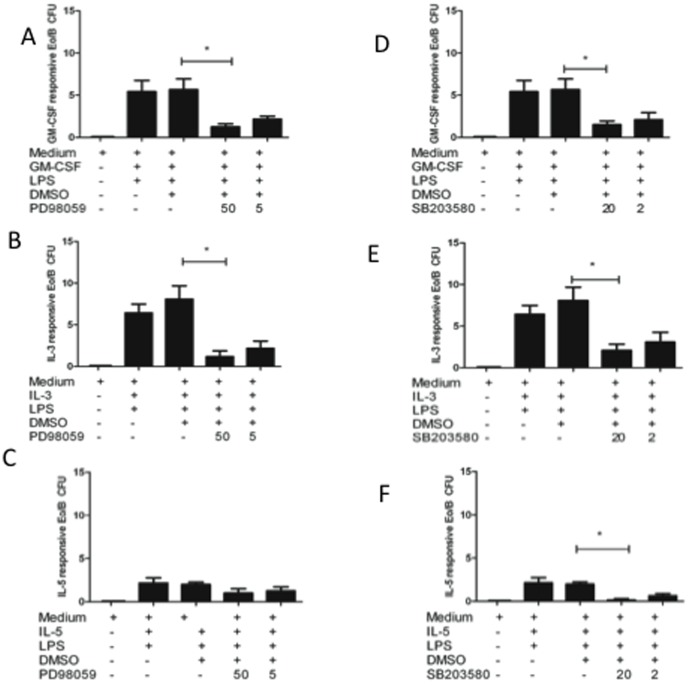
Inhibition of ERK 1/2 decreases GM-CSF- and IL-3-responsive Eo/B CFU, but not IL-5-responsive colonies. CD34^+^ cells were incubated with a pharmacological inhibitor to A–C: ERK1/2 (PD98059) or D–F: p38 MAPK (SB203580) or DMSO vehicle control in methylcellulose cultures stimulated hematopoietic cytokines (A, D) GM-CSF (10 ng/mL), (B,E) IL-3 (1 ng/mL), (C,F) IL-5 (1 ng/mL) with LPS for 14 days (37°C and 5% CO_2_). Eo/B cultures are tight, granular clusters of 40 cells or more. Data are presented as mean ±SEM of 6 experiments (B) and significant findings were determined using ANOVA with post hoc Dunnett comparison and presented by an asterisk (*), p<0.05.

Eosinophilopoiesis is a highly regulated process that has been demonstrated to be dependent on ERK signalling of murine BM CD34^+^ cells [21]. Since we found that IL-4 down-regulated LPS induction of ERK 1/2 (Figure 1C), we next assessed the effects of blocking ERK 1/2 in CB CD34^+^ cell Eo/B CFU formation in the presence of LPS. As shown in Figure 4A–B, the addition of PD98059, a specific pharmacological inhibitor of ERK 1/2 signalling [21], resulted in reduced GM-CSF- (p = 0.009) and IL-3-responsive (p = 0.007) Eo/B CFU formation in the presence of LPS. No differences were observed with IL-5-responsive Eo/B CFU (Figure 4C).

We included an assessment of p38 MAPK inhibition, a kinase that is known to be upregulated by LPS in CB CD34^+^ cells [11], and is involved in eosinophil differentiation from cord blood progenitors [22]. As shown in Figure 4F, blocking p38 MAPK in the presence of LPS reduced IL-5- (p = 0.013) responsive Eo/B CFU, along with GM-CSF- (p = 0.015) (Figure 4D) and IL-3- (p = 0.031) responsive (Figure 4E) Eo/B CFU.

## Discussion

There is evidence that maternal cytokines play instructive roles in neonatal immune responses [6], [15]. Interestingly, maternal atopy is often associated with deficient LPS signalling in infant CB [24]–[27], but the factor(s) that influence this relative deficiency are unclear. In the present report, we investigated how a T_H_2 microenvironment could influence CB progenitor cell Eo/B differentiation responses to LPS [10]. We show for the first time that IL-4, but not IL-13, reduces CB progenitor Eo/B differentiation responses to LPS *in vitro*.

The cytokines IL-4 and IL-13 have been shown to influence hematopoietic progenitor cell chemotaxis [19] and Eo/B CFU [20] in human and murine systems, respectively. In these models, IL-13 was found to have an enhancing effect on both migratory responses to stromal-derived factor 1α and on Eo/B differentiation. Our observation that IL-13 did not have an inhibitory effect on Eo/B CFU (Figure 2D–F) is in line with those of others [20] demonstrating promotion of eosinophilopoiesis. Conversely, there are reports in which IL-4 has an inhibitory role in eosinophilopoiesis of CB-derived progenitor cells [28], [29]. Our findings therefore agree and extend these studies by demonstrating an inhibitory effect of IL-4 on LPS-induced Eo/B CFU (Figure 2A–B), which is dependent on signalling through IL-4Rα (Figure 3A–B) and not IL-13Rα1 (Figure 3D–E). Our results demonstrate that IL-4 and IL-13, which have some redundancy in signalling due to shared receptor subunits, can induce divergent responses when they ligate their cognate receptors [30]. Therefore, non-redundant effects on Eo/B CFU formation result from Type I (IL-4Rα:γc) signalling by IL-4, as opposed to Type II (IL-4Rα:IL-13Rα1) signalling.

It has recently been shown that maternal allergic history is associated with impaired LPS-induced phosphorylation of p38 MAPK and ERK 1/2 in peripheral blood monocytes [24], [26]. Although these molecules can be induced by LPS stimulation in CB CD34^+^ cells [11], we did not assess the effect of allergic risk in our previous study, but focused on the biological effect of LPS stimulation of these cells [11]. Our previous findings that atopic-risk infants produce fewer LPS-induced Eo/B CFU, and stimulation of non-atopic mononuclear cells with IL-4 inhibits LPS signalling, much like that found in the cells of the atopic counterparts [24] add credence to our use of IL-4 to recapitulate the maternal allergic microenvironment *in utero* [12], [13]. Therefore, our present findings suggest IL-4, which can be found in the CB of at-risk infants who subsequently go on to develop disease [12], [13] has an inhibitory effect on Eo/B CFU [28], [29] through altered CB CD34^+^ cell responsiveness to LPS [10].

The use of pharmacological inhibitors in the methylcellulose assay demonstrated the biological importance of IL-4 inhibition of ERK 1/2 signalling in CB CD34^+^ cells. Although inhibition of p38 MAPK resulted in overall decreases in Eo/B CFU derived from stimulation by three hematopoietic cytokines–findings observed by others [22] – it was the inhibition of ERK 1/2 in the cultures that resulted in the specific inhibition of GM-CSF- and IL-3-responsive Eo/B CFU. Our colony data (Figure 4) not only reinforce the importance of MAPK signalling in eosinophil differentiation [21], but also corroborate our phospho-flow analysis illustrating reduced ERK 1/2 expression after IL-4 stimulation (Figure 1C). The addition of IL-4 to the cultures not only reduced both GM-CSF- and IL-3-responsive Eo/B CFU in the presence of LPS (Figure 2A–B), but reduced LPS-induced ERK 1/2 signalling (Figure 1C), a finding supported by others [24]. Furthermore, *in vitro* inhibition of ERK 1/2 significantly reduced both GM-CSF- and IL-3-responsive Eo/B CFU (as opposed to IL-5 responsive colonies), which reinforces the specificity for ERK signalling in IL-4 inhibition of Eo/B CFU formation.

Our *in vitro* work may suggest mechanistic underpinnings for other *in vitro* [31] and *in vivo* [32]–[34] observations in relation to these T_H_2 cytokines and eosinophil counts. It has been shown that patients with atopic dermatitis, a T_H_2- driven disease, have significantly greater peripheral blood eosinophil apoptosis in response to IL-4 [31]. In connection with our findings presented herein, this may suggest that the inhibitory effect of IL-4 on eosinophil survival ranges from immature eosinophil progenitor cells to the mature granulocyte. Preclinical models show success of *in vivo* blockade with IL-4, whereas a convincing effect on eosinophil numbers in humans has been disappointing. Studies by Wenzel et al., [32], [33] and Gauvreau et al., [34] have shown that blocking IL-4Rα improves asthmatic FEV_1_ responses, but has no effect on sputum eosinophils. Given that IL-4 inhibits hematopoietic progenitor cell differentiation into eosinophils (Figure 2A–B) through IL-4Rα (Figure 3A–B), blockade of IL-4Rα (and subsequent blockade of IL-4 signalling) may result in the ability of these progenitor cells found in the bronchial mucosa of asthmatics [35] to differentiate, associated with a lack of reduction in airway eosinophil counts.

The seemingly paradoxical finding that IL-4, a pro-allergic cytokine, reduces LPS-induced Eo/B CFU, may relate to reports suggesting an inverse relationship between eosinophilia and particular ratios of T_H_2 cytokines in allergic diseases [31]. For example, in patients with atopic dermatitis, acute lesions are associated with increased IL-4 mRNA with lymphocytic infiltration, whereas chronic lesions have a predominance of IL-5 mRNA with eosinophil infiltration [36]. Additionally, intrinsic asthma denoted by normal IgE and IL-4 levels, is associated with increased eosinophilia and IL-5 levels, compared to those with extrinsic asthma characterized by increased IL-5 and IL-4 [37]. Therefore, not only may higher IL-4 levels at the site of inflammation be related to reduced eosinophilia in allergic tissue [36], [37], but, *in utero* it may contribute to potential gene (e.g., atopy/IL-4) by environment (e.g., LPS) interactions which influence neonatal progenitor cell Eo/B CFU formation. Therefore, *in utero* or inflammatory tissue site T_H_2 cytokine milieu may play a role in determining the fate of eosinophils and their progenitors.

Differential responses of CB CD34^+^ cells between low- and high-atopic risk neonates may depend on cytokine influences *in utero*, suggesting that an *in utero* atopic (T_H_2) cytokine milieu can influence the innate immune responses of neonatal CD34^+^ cells [10] and thus the development of allergic inflammatory responses in early life.
